# Monitoring cortical neuronal activity and spreading depression in freely behaving familial hemiplegic migraine Cacna1a R192Q knockin mice

**DOI:** 10.1186/1129-2377-14-S1-P79

**Published:** 2013-02-21

**Authors:** T Houben, R Shyti, Q Dees, S van Berloo, L de Groote, GM Terwindt, MD Ferrari, EA Tolner, AM van den Maagdenberg

**Affiliations:** 1 Department of Neurology, Leiden University Medical Center, Leiden, Netherlands; 2 Department of Human Genetics, Leiden University Medical Center, Leiden, Netherlands; 3 Instrument Development Department, Leiden University Medical Center, Leiden, Netherlands

## Introduction

Experimental findings from transgenic migraine mouse models that carry a human FHM1 gain-of-function mutation in CaV2.1 (P/Q-type) calcium channels underscore the role of neuronal hyperexcitability in migraine [1,2]. However, functional data that link the excitability changes to neuronal network activity and the enhanced propensity to cortical spreading depression (CSD), the likely mechanism underlying migraine aura, are largely lacking.

## Purpose/background/objectives

Here, we aimed to set up an electrophysiology platform to study changes in cortical neuronal network activity in relation to CSD in freely behaving transgenic migraine mice.

## Methods

We developed an electrophysiology system for long-term recordings of DC-EEG and multi-unit-activity from the cortex of freely behaving mice. The system combines a counterbalanced 7 channel swivel with custom-built differential DC-EEG, AC-EEG and unit activity amplifiers. Stable DC-EEG recordings are obtained using AgAgCl epidural electrodes, while intracortical platinum electrodes are used for simultaneous recording of multi-unit-activity and AC-EEG. For CSD induction intracortical microdialysis was used for infusion of high KCl solution.

## Results

Simultaneous recordings of multi-unit activity, DC- and AC-EEG were made from the sensorimotor and occipital cortex of wild-type and FHM1 migraine mice for up to 3 weeks. Apart from spontaneous cortical activity, visual evoked cortical responses were induced using 1 ms blue light pulses. Microdialysis with KCl solution resulted in successful induction of CSD events in the awake mice.

## Conclusion

We established a novel platform for performing longitudinal recordings of cortical neuronal activity and spreading depression in freely behaving mice carrying migraine-specific mutations. Using this platform, we aim to characterize how cortical activity is altered by modulatory factors that predispose for migraine attacks.
